# ERAD defects and the HFE-H63D variant are associated with increased risk of liver damages in Alpha 1-Antitrypsin Deficiency

**DOI:** 10.1371/journal.pone.0179369

**Published:** 2017-06-15

**Authors:** Philippe Joly, Hélène Vignaud, Julie Di Martino, Mathias Ruiz, Roman Garin, Lioara Restier, Abdelouahed Belmalih, Christelle Marchal, Christophe Cullin, Benoit Arveiler, Patricia Fergelot, Aaron D. Gitler, Alain Lachaux, Julien Couthouis, Marion Bouchecareilh

**Affiliations:** 1 University Lyon - University Claude Bernard Lyon 1 - EA 7424 – Inter-university Laboratory of Human Movement Science, Villeurbanne, France; 2 Laboratoire de Biochimie et biologie moléculaire Grand-Est, Hôpital Edouard Herriot, Hospices Civils de Lyon, Lyon, France; 3 CNRS, University Bordeaux, UMR5095 Institut de Biochimie et Génétique Cellulaires, Bordeaux, France; 4 INSERM, University Bordeaux, UMR1053 Bordeaux Research In Translational Oncology, BaRITOn, Bordeaux, France; 5 Department of Paediatric Gastroenterology, Hepatology and Nutrition, Children's Hospital of Lyon, Lyon, France; 6 University Bordeaux, INSERM U1211, Laboratoire Maladies Rares, Génétique et Métabolisme (MRGM), Bordeaux, France; 7 Department of Genetics, Stanford University School of Medicine, Stanford, California, United States of America; Louisiana State University Health Sciences Center, UNITED STATES

## Abstract

**Background:**

The most common and severe disease causing allele of Alpha 1-Antitrypsin Deficiency (1ATD) is Z-1AT. This protein aggregates in the endoplasmic reticulum, which is the main cause of liver disease in childhood. Based on recent evidences and on the frequency of liver disease occurrence in Z-1AT patients, it seems that liver disease progression is linked to still unknown genetic factors.

**Methods:**

We used an innovative approach combining yeast genetic screens with next generation exome sequencing to identify and functionally characterize the genes involved in 1ATD associated liver disease.

**Results:**

Using yeast genetic screens, we identified HRD1, an Endoplasmic Reticulum Associated Degradation (ERAD) associated protein, as an inducer of Z-mediated toxicity. Whole exome sequencing of 1ATD patients resulted in the identification of two variants associated with liver damages in Z-1AT homozygous cases: HFE H63D and HERPUD1 R50H. Functional characterization in Z-1AT model cell lines demonstrated that impairment of the ERAD machinery combined with the HFE H63D variant expression decreased both cell proliferation and cell viability, while Unfolded Protein Response (UPR)-mediated cell death was hyperstimulated.

**Conclusion:**

This powerful experimental pipeline allowed us to identify and functionally validate two genes involved in Z-1AT-mediated severe liver toxicity. This pilot study moves forward our understanding on genetic modifiers involved in 1ATD and highlights the UPR pathway as a target for the treatment of liver diseases associated with 1ATD. Finally, these findings support a larger scale screening for HERPUD1 R50H and HFE H63D variants in the sub-group of 1ATD patients developing significant chronic hepatic injuries (hepatomegaly, chronic cholestasis, elevated liver enzymes) and at risk developing liver cirrhosis.

## Introduction

The failure of proteins to properly exit the endoplasmic reticulum results in a large number of diseases [1]. One such protein is the normally soluble luminal protein Alpha 1-Antitrypsin (1AT). As a member of the serine protease inhibitor (serpin) family, 1AT is also the most abundant antiprotease in the serum [2]. This protein is mainly produced by hepatocytes in the liver to act as a general inhibitor of serine proteases [3]. From here it will be transported by the bloodstream to the lung where its activity is critical for the inhibition of proteases that degrade elastin, a key component of the extracellular matrix responsible for the elastic properties of the lungs [4, 5]. Therefore, hepatic production of 1AT is necessary for maintaining the protease/anti-protease balance in the serum [6, 7].

Several 1AT mutations have been previously characterized and have been shown to prevent the 1AT protein from properly exiting the ER, thus impairing its secretion. This results in Alpha 1-Antitrypsin Deficiency (1ATD) [6, 7].

The most severe and common disease causing allele in 1AT is called "Z variant", which is caused by a single Glu342Lys amino acid substitution [8–10]. This point mutation not only leads to inefficient export of soluble monomers from the ER, but also in the accumulation of Z-1AT protein aggregates in the ER. About 8% of the population homozygous for the Z variant (ZZ) develops a clinically significant liver disease, associated with intracellular accumulation of Z aggregates that can progress to end-stage pathology requiring liver transplantation [8, 9]. There is a considerable variability in the age-of-onset and severity for this liver disease component associated with 1ATD, and as of today, it is still impossible to predict which individuals with 1ATD may develop severe liver disease.

Such a high variation in the severity of Z-1ATD-associated liver damages suggests that genetic modifiers or environmental factors may be risk and age-of-onset modifiers. In 2009, a candidate gene-sequencing strategy demonstrated that differences in endoplasmic reticulum mannosidase I (ERmanI) expression were associated with an earlier age-of-onset for end-stage liver disease [11]. However, the significance of this association has been challenged, as a replication cohort failed to reproduce the results [12]. Even if the ERmanI polymorphism does not prove to be a clinically significant modifier of 1ATD, many other factors are likely to be able to modify 1ATD-associated liver disease [13]. Given that liver transplantation is currently the only therapeutic strategy available for the severe liver disease caused by 1ATD, it is therefore critical to identify which genes are involved in Z-1AT-mediated liver toxicity. Validating the functional impact of such genes would furthermore allow their use as biomarkers and would likely improve disease outcome and clinical handling of the patients.

In this study, we performed yeast genetic screens and whole exome sequencing (WES) of small Z-1ATD families to identify genes involved in Z-1AT-mediated liver toxicity. Based on this experimental pipeline and subsequent functional validations in relevant disease models, we identified two mutations, HERPUD1 R50H and HFE H63D that were associated with the advanced liver disease component of 1ATD. These two variants highlighted specific pathways, including Endoplasmic Reticulum Associated Degradation (ERAD) and the Unfolded Protein Response (UPR), which could represent novel risk factors for 1ATD-caused liver disease.

## Materials and methods

### Materials

Tunicamycin was purchased from Calbiochem (Merck Bioscience, Darmstadt, Germany) and was dissolved in DMSO to a final stock concentration of 10 mg/ml. SiRNA were obtained from Ambion (Austin, TX, USA). Rabbit anti-human HERPUD1 antibody was purchased from Abcam (Cambridge, MA, USA). Rabbit anti-human SYVN1 was purchased from Cell Signaling (Danvers, MA, USA). Mouse anti-human Hsp90, and goat anti-human BiP antibodies were purchased from Santa Cruz Biotechnology (Santa Cruz, CA, USA). Cycloheximide and Flag tag antibody were purchased from Sigma (St Louis, MO, USA). Mouse anti-human GAPDH was purchased from Genetex (Irvine, CA, USA). Human 1AT antibody was purchased from Immunology Consultants Laboratory (Newberg, OR, USA).

### Yeast strains, media and plasmids

To screen for yeast KO strains involved in Z-1AT-mediated toxicity, the haploid α-mating-type (BY4742) *S*. *cerevisiae* deletion mutant collection was obtained from the Euroscarf yeast deletion strain set [14]. Plasmids (empty vector, Wild-type 1AT and Z-mutant 1AT expression plasmids) were introduced into pooled strains by lithium acetate transformation [15]. All yeast transformations were carried out as described previously [16]. As specified, yeasts were grown in SD medium (0.67% yeast nitrogen base, 2% dextrose) to repress 1AT expression or SG medium (0.67% yeast nitrogen base, 2% galactose) to induce 1AT expression. All media were supplemented with 20 mg/L histidine (H), 20 mg/L lysine (K), and 60 mg/L leucine (L). Yeast expression plasmids were generated by Gateway recombination (Invitrogen, Carlsbad, CA, USA) using pAG426-ccdB as the destination plasmid [17], a multicopy (*2μ*) yeast expression plasmid with an *URA3* selectable marker and a *GAL1* promoter, as previously used for such studies [18]. The pAG426-ccdB empty plasmid was also used as control/empty vector for cell viability studies. Gateway donor plasmids were generated for 1AT constructs by PCR amplification of WT-1AT and Z-1AT human cDNA using the Phusion^®^ Taq High Fidelity DNA Polymerase (Thermo Fisher Scientific- Rockford, IL, USA).

### Colony immunoblot assay

To assay 1AT secretion by yeast colony immunoblot, cells expressing 1AT were spotted onto nitrocellulose. After 48 h of growth at 30°C cells were rinsed from the nitrocellulose with distilled water and then immunoblotted for 1AT as described below (Immunoblotting).

### Spotting assay

All spotting assays were performed in the same conditions. Tenfold serial dilutions starting with equal number of cells (10^7^ cells) were made in sterile water. Spotting assays derived from a pool of three independent fresh colonies. Five microliters drops were then plated on SD (expression repressed) or SG medium (expression induced) complemented with appropriated amino acids.

### Cell culture, transfections and treatments

Wild-type (also named M) and Z-variant IB3 cells were cultured in LHC-8 medium containing 200 μg/ml G418 [19]. The different silencing using siRNA were performed with RNAiMax (Invitrogen, Carlsbad, CA, USA) as per the manufacturer’s protocol using 50 nM of the indicated siRNA. Wild-type and Z-variant IB3 cells were transiently transfected with the pcDNA-Herp and mutants or H63D plasmid. Transfection was done using Lipofectamine 2000 (Invitrogen, Carlsbad, CA, USA) according to the manufacturer's recommendations.

### DNA constructs

Human HFE was PCR amplified and cloned into the NHEI and NotI sites of the pcDNA3.1(+) expression plasmid. The pcDNA3-HFE-H63D construct was obtained as described previously [20, 21]. The pcDNA3-HERPUD1-WT construct was a gift of Dr Lederkremer [22]. The K61R and R50H mutations were obtained as previously described [23].

### siRNA-treatment

Wild-type and Z-variant IB3 cells were plated in 12 or 384-wells plates and grown to 30–40% confluence. Silencing of individual target was performed as indicated above and as described previously [19, 24, 25].

### Immunoblotting

Cell lysates were prepared in 50 mM Tris-HCl, 150 mM NaCl, 1% (v/v) Triton X-100 with protease inhibitors and protein concentrations determined by Bradford protein assay (Thermo Scientific, Rockford, IL, USA). Total protein extract was re-suspended in 1X SDS sample buffer containing DTT and incubated at 95°C for 5 min. For each sample 10–25 μg of total protein was separated on a 10% (v/v) SDS-PAGE then transferred to nitrocellulose and immunoblotted with indicated primary antibodies. Detection was performed using chemiluminescence and the appropriate horseradish peroxidase (HRP)-conjugated secondary antibodies.

### 1AT secretion assays

3 h prior to measurement of 1AT secretion kinetics, cells were washed with PBS and incubated with 350 μl (12-well plate) of FBS-free culture medium. After the 3 h incubation, cells were harvested, and the corresponding media was centrifuged at 1500 rpm for 30 min at 4°C to separate cells and medium. Following cell lysis, quantification of immature and mature forms of 1AT respectively in the cell lysate or secreted into the culture media was performed as described previously [19].

### Soluble and insoluble fractions from cell lysates

Cell lysates were prepared and soluble and insoluble fractions were obtained as previously described [26, 27].

### Viability assay

The WT-IB3 and Z-IB3 cells were cultivated for 5 days in a 384-wells plate format. Two siRNA treatments (at day 2 and day 4) were performed during these 5 days. At day 5, the cells were tested on viability using ApoTox-Glo^™^ Triplex assay and CellTiter-Glo Luminescent Cell Viability Assay Promega (Wisconsin, USA) according to the manufacturer's recommendations.

### Study approval

The protocol of the exome sequencing has been approved by the "Comité de protection des personnes Sud Est IV" (N°ID RCB: 2012-A00204+39; Protocole HCL/P 2011–663). DNA from patients was obtained according to standard protocols and written informed consent was obtained from all participants or their legal guardians.

### Count of viable cells

Viable cells number was determined using a TC20^™^ Automated Cell Counter (BIO-RAD) according to the manufacturer′s protocol.

### Data analysis

The error bars represent the SEM (*n*≥3) of at least 3 independent experiments or the SD of the mean. In all panels asterisks indicate a *p*-value <0.05 as determined by a two-tailed *t*-test using the control as the reference. The values represent densitometric analysis of Western blots using ImageJ software (http://imagej.nih.gov). In all cases, the densitometry was performed below the saturation limit of the antibody.

### Quantification of 1AT glycoforms

Immunoblot exposures shown in the Results were selected to allow visualization of 1AT under identical protein loads in same SDS-PAGE for all treatments. Given the dynamic range, quantitation of the band Immature (I), Mature (Mat) and Secreted (S) glycoforms were made by analysis of band intensities that were in the linear range. In brief, the X-ray films were exposed for an increasing amount of time and the different exposures were quantified using ImageJ densitometer/software package (http://imagej.nih.gov). When Immature (I) and Mature (Mat) bands were quantified from different exposures an internal reference was then used to normalize the signal intensity (Hsp90 or GAPDH).

### Statistical analysis

All experiments were performed in at least 3 independents replicates. Student t-test was used for most comparisons. Kinetic curves were analyzed by two-way ANOVA followed by Bonferroni post-test using the Prism software application (Version 6, GraphPad, San Diego, CA).

### Exome sequencing

Genomic DNA samples were isolated from family members using Oragene DNA collection kit (DNA Genotek Inc. Ottawa, Ontario, Canada). DNA samples were sent to GATC (GATC Biotech AG, Konstanz, Germany) and IGBMC Microarray and Sequencing Platform (ILLKIRCH, France) to generate the exome library using the SureSelect Human All Exon 50 Mb kit (Agilent, Santa Clara, CA). Sequencing was performed with 200 bp paired-end reads on an Illumina HiSeq platform (Illumina Inc., San Diego, CA). Reads were aligned to the human reference genome (UCSC hg19, GRCh37, Feb. 2009 release) using bowtie2 and SAMtools. We applied GATK base quality score recalibration, indel realignment, duplicate removal, and performed coverage calculations. SNP and INDEL discovery and genotyping across each sample was performed using variant quality score recalibration. Variants were filtered against dbSNPv137, 1000 genomes and ESP 6500 databases and were then annotated using ANNOVAR [28]. All bioinformatics analyses were performed on Sherlock, a Stanford HPC cluster.

## Results

We used two complementary approaches: yeast screens and next generation exome sequencing to identify new genes involved in 1ATD-associated liver damages.

### Genome-wide toxicity screen in yeast

To date, a whole genome analysis to identify genetic factors involved in Z-1AT-mediated toxicity has not been undertaken. For the vast majority, mammalian model cell lines used in the study of 1ATD derivate from immortalized and/or transformed cell lines, impeding our ability to analyze genetic factors able to induce toxicity. To overcome this limitation, we decided to perform our genome-wide toxicity screen in the baker’s yeast *S*. *cerevisiae*. Simple model organisms, such as yeast, have previously been used successfully to identify new genes involved in human diseases and complex pathologies involving protein aggregation [29–32].

**Fig 1:**
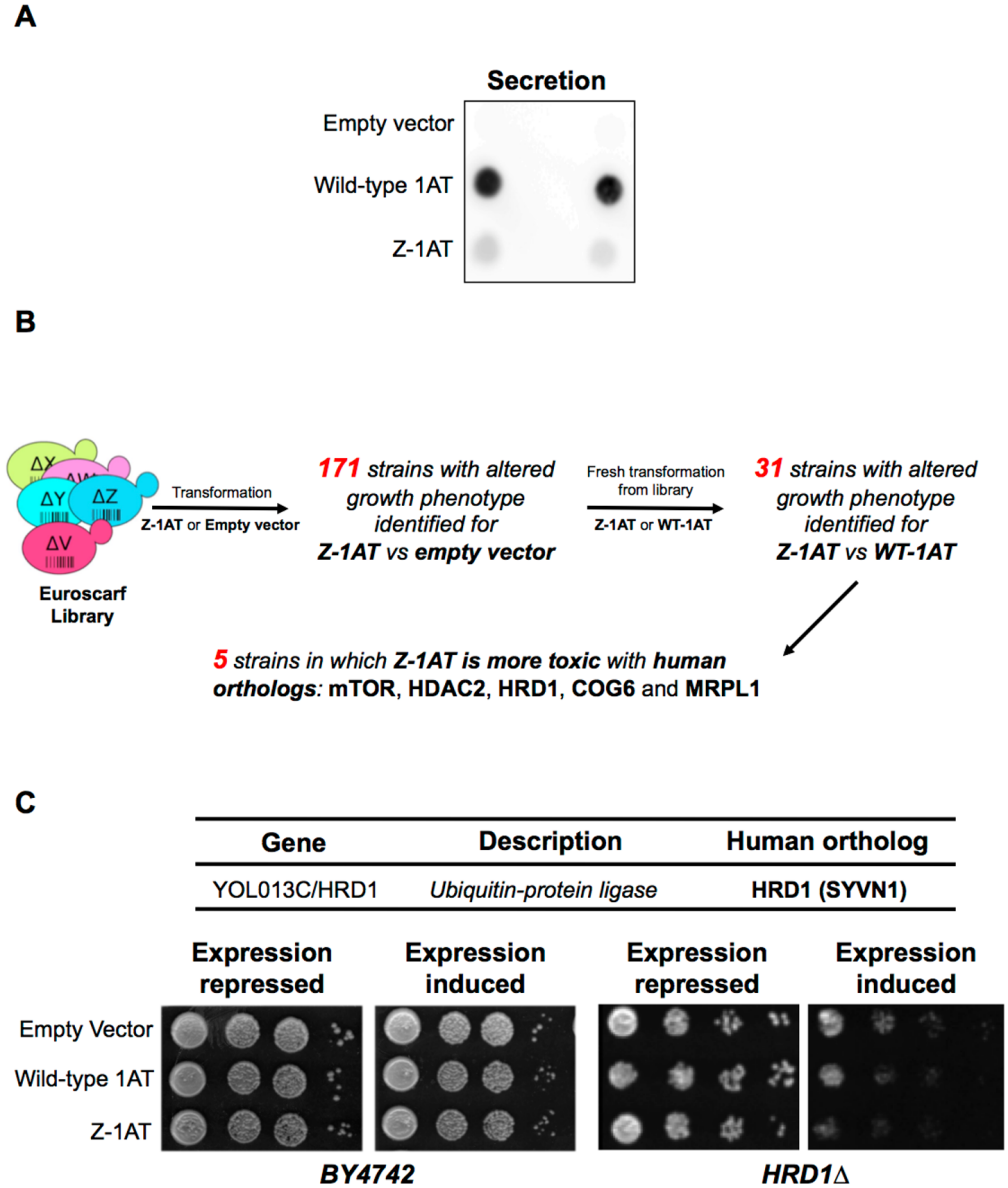
Identification of HRD1 as an important actor in Z-1AT-mediated toxicity in a toxicity assay set up in *S*. *cerevisiae*. (**A**) Empty Vector, Wild-type (WT) 1AT and Z-1AT expressing cells were grown in inducible media and spotted onto nitrocellulose. After 48 hr of growth at 30°C, the cells were rinsed from the nitrocellulose with distilled water and then immunoblotted for 1AT. (**B**) Yeast screen methodology summary. The expression of Z-1AT is not toxic in yeast. As a consequence we proposed to identify genes whose presence is necessary for this absence of toxicity, the latter being revealed upon target gene deletion. The Euroscarf library of individual gene deletion mutants is transformed by expression plasmids for either Z-1AT, WT-1AT or the empty vector. Individual colonies are picked and plated in 96-well plates. The growth test is then initiated by inducing the expression of the recombinant 1AT proteins on specific media. Clones with defective growth are selected and the DNA barcode sequence for gene identification purposes. In brief, through this screen, we identified 171 KO strains in which Z-1AT toxicity was slightly changed compared to the empty vector. Then, we have tested these 171 KO strains for the toxicity induced by the WT-1AT expression and found 31 KO mutants in whom Z-1AT was more toxic than the WT-1AT. Finally, only 5 genes displayed a clear human ortholog. (**C**) Yeast cells transformed with empty vector, WT-1AT or Z-1AT were grown in liquid synthetic complete medium to log phase. Cells were spotted on plates containing SD medium (2% dextrose) to repress 1AT expression or SG medium (2% galactose) to induce 1AT expression, and were incubated at 30°C for 3 days. Shown is fourfold serial dilution starting with equal numbers of cells. Spotting assays for *HRD1* deletion strain (right panels) and the parental control strain (BY4247 –left panels) are shown.

In order to screen genetic factors involved in Z-1AT-mediated toxicity, we used a previously published yeast 1AT expression system [18]. Galactose inducible yeast expression plasmids were generated for Z-1AT and Wild-Type (WT)-1AT (see Material and methods). This inducible system drives rapid expression of the human WT and Z-1AT while yeast are grown in the presence of galactose in the media. In humans, WT-1AT is produced and secreted by the hepatocytes into the bloodstream. Conversely, the Z variant is retained into the ER and poorly secreted. Using this yeast expression system in a comparative secretion efficiency test evaluated by immunoblot analysis of conditioned (in presence of galactose) media, only WT-1AT was detectable in the extracellular media (Fig 1A). Similarly, we confirmed by spotting assays that yeast expression of WT or Z-1AT proteins did not present any related toxicity [33, 34] (Fig 1C-left, BY4742). This feature allowed us to perform genetic screens to identify modifier genes that unmask or enhance Z-1AT toxicity.

This genome-wide yeast screen was based on the EuroScarf collection of null mutants of *S*. *cerevisae*. This collection contains 4850 barcoded viable mutants [35], each lacking a single gene (Fig 1B) [36]. All these Knock-Out (KO) strains were pooled and the plasmids containing the human Z-1AT, WT-1AT or the empty vector were introduced by transformation [37] and clones were selected on dextrose media. Strains showing Z-1AT toxicity were identified by comparison to the control strain (empty vector) present on the same plate. Of 4850 mutants, we have identified 171 KO strains in which Z-1AT expression is deleterious compared to an empty vector (Fig 1B).

As we wanted to isolate strains in which Z-1AT expression leads to an alteration in the yeast growth compared to the WT-1AT, we performed a second screen using these 171 KO strains. Strains, in which Z-1AT expression is deleterious, while WT-1AT is not, were selected as previously described. This second screen allowed us to narrow down our candidates to 31 KO mutants in whom only Z-1AT altered the growth of strains compared to both empty and WT-1AT expressing constructs (Fig 1B). Among these 31 mutants, only five of the yeast genes had human orthologs (Fig 1B and S1 Fig). Although we expected more candidates with a strong growth defect, all mutant strains that enhanced Z-1AT toxicity had a phenotype that was significantly more severe than that observed in yeast expressing WT-1AT (Fig 1C). Human orthologs of those KOs were HDAC2 (Histone Deacetylase 2), MRPL1 (Mitochondrial Ribosomal Protein L1), MTORC1 (Mechanistic Target Of Rapamycin Complex 1), COG6 (Component of Oligomeric Golgi complex 6) and HRD1 (HMG-CoA Reductase Degradation 1 homolog) (Fig 1B and S1 Fig). Remarkably, HRD1 and HDAC2 have already been shown to be involved in Z-1AT trafficking and regulation of its ER accumulation/degradation [19, 38], validating our data and confirming the efficacy of our strategy.

### HRD1 is involved in Z-1AT toxicity

**Fig 2:**
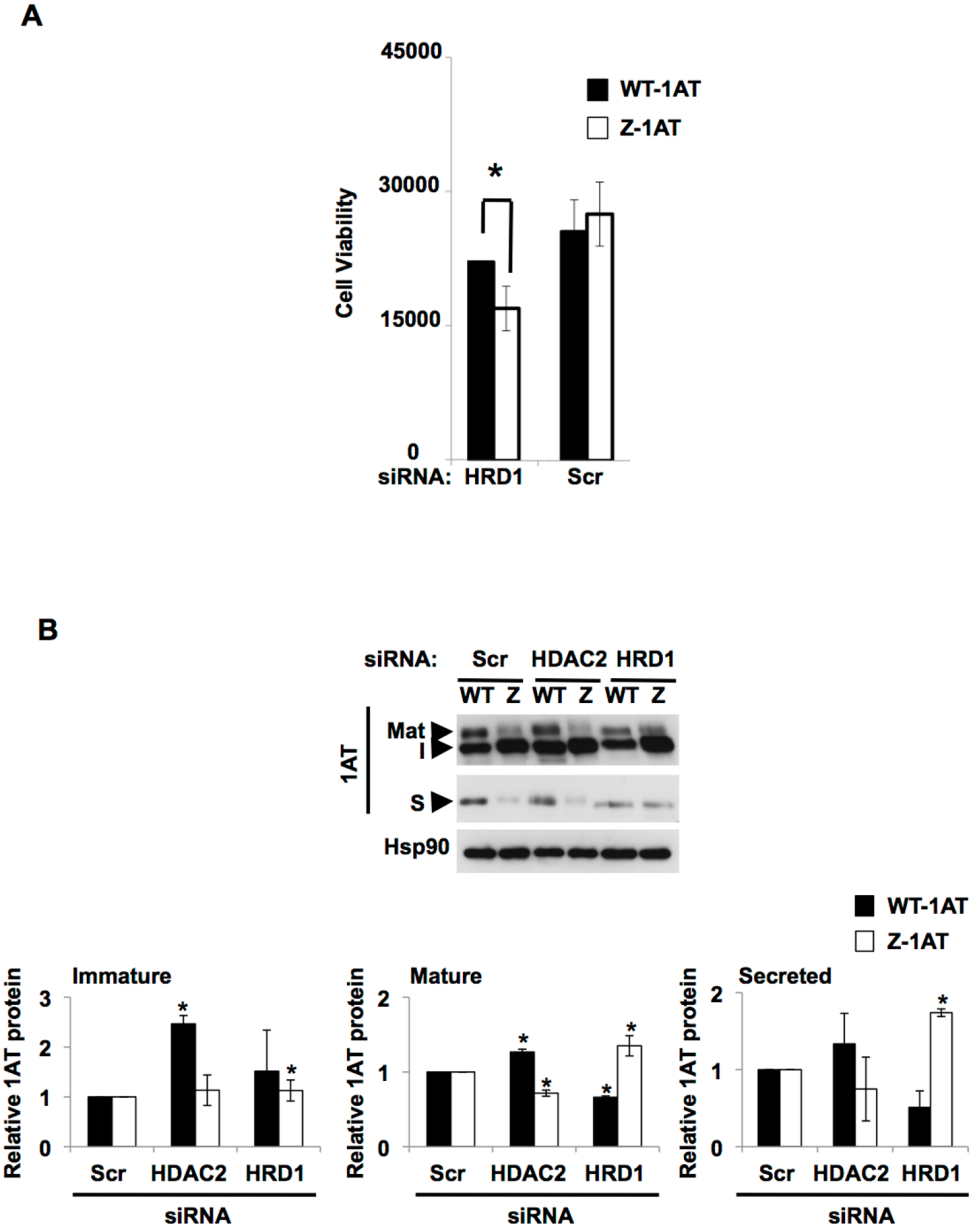
HRD1 silencing affects the viability, the traffic and secretion of human cells lines expressing the Z variant. (**A**) Viability is measured using GF-AFC Substrate. This compound can enter live cells where it is cleaved by the live-cell protease to release AFC. WT and Z-IB3 cells were treated as described in Material and methods. Quantitative analysis of candidate genes silencing on viability in WT or Z-IB3 cells (mean ± S.D., n = 3 independent experiments). The * indicates p < 0.05 as determined by two-tailed t-test using WT-IB3 cells as reference. (**B**) Immunoblot analysis of 1AT and Hsp90 protein expression in cell lysates and culture media following siRNA-mediated silencing of the candidate genes HDAC2 and HRD1 in WT and Z-IB3 cells. Traffic of the 1AT glycoprotein through the secretory pathway can be monitored by a change in its migration on SDS-PAGE in response to the processing of ER-acquired N-linked oligosaccharides (the immature form: I) during trafficking through the Golgi to generate the slower migrating, mature glycoform (Mat). The latter is secreted in the serum (secreted form: S) by the cell. Quantitative analyses of the immature, mature, and secreted forms of WT-1AT (black bar graph) and Z-1AT (white bar graph) after candidate genes silencing relative to scrambled control (Scr). Data denote the -fold change in the protein expression of the indicated WT or Z-1AT forms relative to Scr (mean ± S.D., *n* = 3 independent experiments). In all panels, the * indicates *p* < 0.05 as determined by two-tailed t-test using Scr as reference.

We next investigated whether these 5 candidate genes isolated from our yeast screen might be involved in 1ATD associated liver disease in human model cell lines [19]. To this end we monitored the toxicity of Z-1AT upon silencing of these 5 genes in relevant human cell lines, using two high throughput assays (Cell titer Glo and ApoTox-Glo Triplex^™^Assay), [19]. We observed an increase of Z-1AT toxicity compared to WT-1AT following HRD1 silencing (Fig 2A and S2 Fig). Consistent with a role of HRD1 in Z-1AT toxicity, only its silencing resulted in a major change in Z-1AT trafficking when compared to any of the other candidate genes (Fig 2B and S3 Fig). As previously observed, HRD1 silencing induced an increase in the immature (ER forms), mature, and secreted pools of Z-1AT (Fig 2B) [38].

### HRD1 and Unfolded Protein Response

HRD1/synoviolin (SYVN1) is involved in the ER-Associated Degradation (ERAD) mechanism. More precisely, HRD1 is a major ubiquitin ligase (E3) that retrotranslocates and ubiquitinates ERAD substrates [39]. One of HRD1 ERAD clients is the Z mutant. It has been previously shown that HRD1 interacts with the Z-1AT mutant. HRD1 can increase the solubility of Z-1AT and facilitate its degradation through ERAD. HRD1 enhances the removal of Z-1AT through ERAD and attenuates intracellular Z-1AT accumulation and toxicity [38]. Conversely, HRD1 knockdown stabilizes the Z-1AT misfolded protein levels in the ER, especially the insoluble fraction or aggregate form [38].

**Fig 3:**
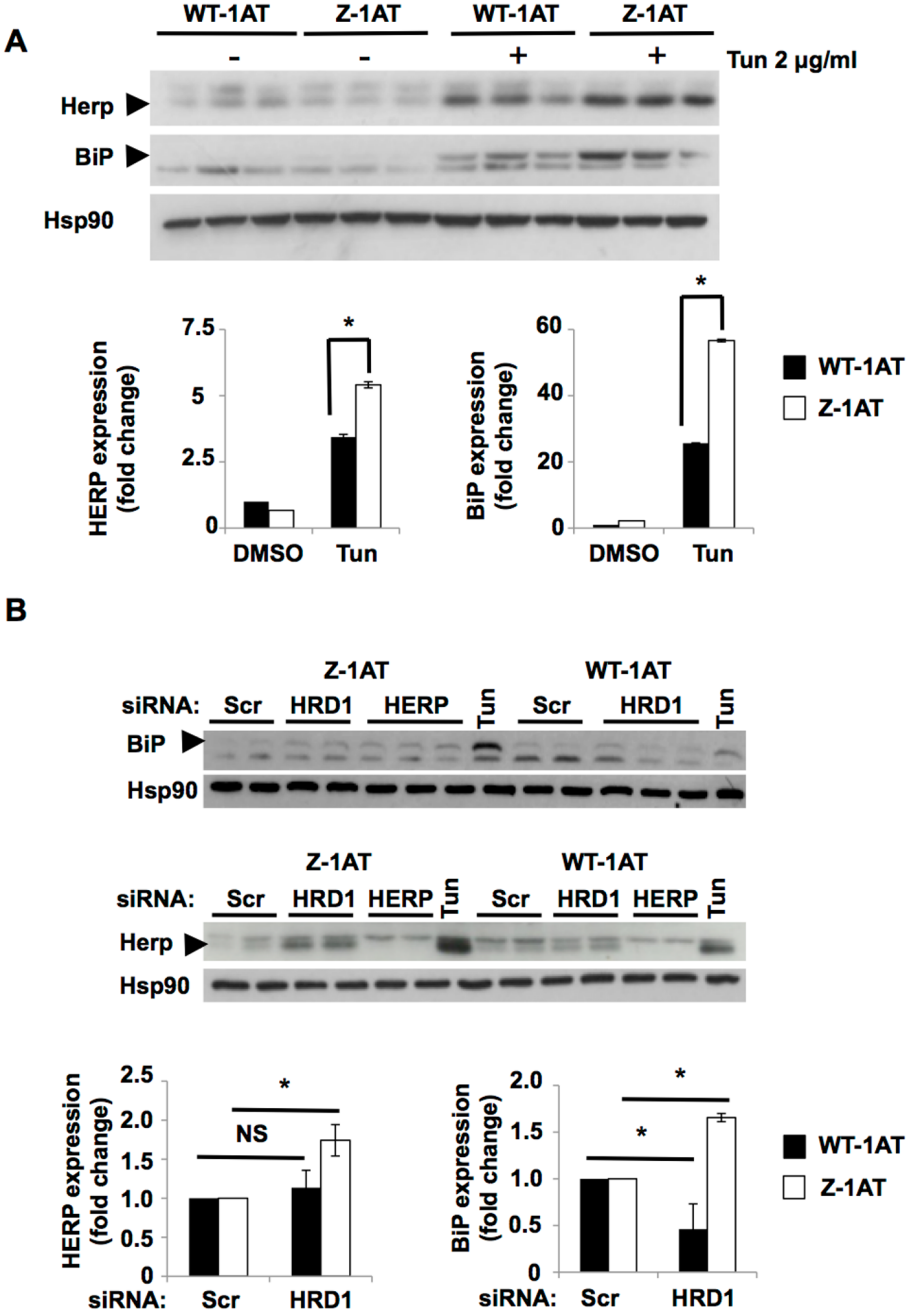
HRD1 silencing increased sensitivity to UPR in cells expressing the Z mutant. (**A**) Immunoblot of HERPUD1 (Herp), BiP and Hsp90 protein expression in cell lysates following treatment of WT and Z-IB3 cells with vehicle or 2 μg/ml Tunicamycin (Tun) for 24 hours. Quantification of Herp and BiP protein expression following vehicle or Tun treatments as indicated at the bottom of the graphs. Data are presented as ratio relative to control (mean ± SD, n = 3 independent experiments). The asterisk indicates p < 0.05 as determined by two-tailed t-test using WT-IB3 cells as the reference. Arrowheads indicate Herp and BiP expression bands. (**B**) Immunoblot of HERPUD1 (Herp) (lower), BiP (upper) and Hsp90 protein expression in cell lysates following siRNA-mediated silencing of Herp or HRD1 in WT and Z-IB3 cells. Quantification of Herp and BiP protein expression following HRD1 siRNA treatments as indicated at the bottom of the graphs. Data are presented as ratio relative to Scr (mean ± SD, n = 3 independent experiments). The asterisk indicates p < 0.05 as determined by two-tailed t-test using Scr as reference. Arrowheads indicate Herp and BiP expression bands. NS = No Significant.

Several signaling pathways are activated in response to an overload of misfolded proteins into the ER. For example, the Unfolded Protein Response (UPR) is induced following accumulation of misfolded protein in order to restore the ER homeostasis [40, 41]. This response is regulated by 3 branches: IRE1, PERK and ATF6. Under normal conditions, each sensor is held in an inactive state [40, 41]. When misfolded protein levels increase during ER stress, the UPR is activated through its 3 sensors and induce a transient attenuation of protein translation with enhancement of the ER’s protein-folding capacity and protein degradation [40, 41]. If ER homeostasis is restored following the UPR activation, the survival pathway is favored and the cells can adapt to the stress. Alternatively, if ER stress is prolonged, the death pathway is activated by the UPR [40, 41]. Given the role of the UPR following misfolded protein accumulation leading to cell death, we hypothesized that the alteration of the ER luminal environment following HRD1 silencing might destabilize ER homeostasis in a sufficient manner to trigger the UPR activation. To address this hypothesis, we first monitored the ability of the Z-1AT aggregates to induce the UPR machinery. As expected [42], immunoblotting analysis of protein expression upregulated by the UPR (BiP/GRP78 and HERPUD1) confirmed that Z-1AT aggregates alone failed to activate the UPR. Neither WT-1AT nor Z-1AT induced the UPR (Fig 3A). In contrast, and as previously shown [42], cells expressing the Z-1AT mutant showed a hyperactivation response in UPR activation after treatment with tunicamycin, an ER stress inducer, compared to the WT-1AT (Fig 3A). This effect was also observed by Ordonez *et al*. [42] at different time points and when cells were treated with low concentrations of glucose (10.0–0.2 mM) and tunicamycin (5–25 ng/mL). Remarkably, in our experimental conditions, a similar response was observed when HRD1 was silenced. Immunoblotting analysis for the same UPR markers revealed a statistically significant increase in expression levels of BiP and HERPUD1 between the WT-1AT and the Z-1AT mutant (Fig 3B). The Z-1AT mutant showed a hypersensitive response in UPR activation following impairment in the ERAD machinery. These results suggest that the UPR could be involved in the higher toxicity observed following HRD1 silencing in the cells expressing the Z mutant.

### Exome sequencing and functional studies

In parallel to previous experiments, we performed whole exome sequencing (WES) for 5 small families with patients homozygous for Z-1AT (ZZ), in which some but not all ZZ siblings presented liver damages ("affected") while other ZZ siblings were without hepatic pathology (“unaffected”) (Table 1). Those families were selected from the cohort built on the DEFI-ALPHA project, a multi-centre cohort for the descriptive study of 1ATD infants that aims at characterizing the clinical and biological factors linked to the appearance of liver disease [43, 44]. As described in Table 1, these infants are all homozygous for the Z-1AT mutation and no additional confounding factors were identified (alcohol, hepatitis, obesity…) or iron accumulation. All these ZZ infants’ patients are Caucasian and included 2 females and 9 males. Detailed clinical information for each patient is summarized in Table 1. The affected patients present a broad range of liver symptoms, from a simple increase in liver enzymes (Alanine Aminotransferase (ALT) and Gamma-glutamyl Transferase (GT)) to a liver transplantation history. Two affected patients present severe liver damages. One of them had undergone orthotopic liver transplantation while the other is highly at risk of developing liver cirrhosis. She had already developed a large hepatomegaly (4 cm under mammary and 3.5 cm substernal), a chronic cholestasis and presents a high level of ALT and GT (Table 1). Following the analysis and comparison of the WES data, several novel variants shared between affected but not present in unaffected siblings were identified. Given the small number of patients available for study, we subsequently focused on genes involved in ER homeostasis and on genes previously associated with liver dysfunctions. This filter led us to identify two promising SNPs: one on HERPUD1 (rs2217332; c.G149A; p.R50H) and one on HFE (rs1799945 c.C187G; p.H63D).

**Table 1:** Genotypes of Herp and HFE variants among ZZ individuals who developed or not liver symptoms.

		Sex	Age	Hepatic Status	1AT	HFE	Herp
**Family 1**	Sibling 1	M	16	Cirrhosis, Transplanted at 8	ZZ	×	×
	Sibling 2	M	14	Normal	ZZ		×
**Family 2**	Sibling 1	M	12	Normal	ZZ		×
	Sibling 2	F	5	Increase enzymes level (ALT 102 U/L; GT 21U/L)	ZZ		×
**Family 3**	Sibling 1	M	20	GT > 20 U/L	ZZ	×	
	Sibling 2	M	23	Normal	ZZ		
**Family 4**	Father	M	43	Normal	ZZ		×
	Sibling 1	M	7	Increase in enzymes level (ALT 82 U/L; GT 31U/L)	ZZ		×
	Sibling 2	F	4	Hepatomegaly, cholestasis and high level of enzymes (ALT 152 U/L; GT 131 U/L)	ZZ	×	×
**Family 5**	Sibling 1	M	13	GT > 20 U/L	ZZ		
	Sibling 2	M	10	GT > 40 U/L	ZZ		

5 families matching our criteria were sequenced. The different families are presented in the two first left-hand columns. These patients are all homozygous for the Z-1AT mutation as indicated in the column named “**1AT**” (ZZ means homozygous). Among all the families already sequenced, only one parents was homozygous for the Z-1AT mutation and has been sequenced (Family 4—Father). “**Sex**” (M for Male and F for Female), “**Age**” and “**Hepatic Statuts**” columns present the age, the gender and the hepatic symptoms for all the patients sequenced at the date of this study. The concentration of liver enzymes Alanine Aminotransferase and Gamma-glutamyl Transferase referred to as respectively ALT and GT and used as marker of liver disease are mentioned when their concentration are higher the reference range. The X mark in the right-hand columns named “**HFE**” and “**Herp**” represent the presence of SNPs: HERPUD1 SNP (rs2217332; c.G149A; p.R50H) and HFE SNP (rs1799945 c.C187G; p.H63D) in the corresponding gene.

HFE (High Fe) is a major histocompatibility complex class 1 protein for which mutations have been associated with Hereditary Hemochromatosis, a cellular iron overload [45]. Hepatocytes are the main site of synthesis of HFE and play a major role in the pathophysiology of Hemochromatosis [45]. HFE have two common alleles, C282Y and H63D [45], and while clinical hemochromatosis is mostly associated with C282Y homozygosity while the frequency of H63D alleles is higher in 1ATD patients versus in the general population (42 vs 27%) [46]. Further analysis showed that HFE variants were significantly associated with higher liver iron deposits in cases of 1ATD, but not hepatitis C-related cirrhosis. It also has been shown that patients with abnormal 1ATD alleles and genetic hemochromatosis may progress to cirrhosis sooner than the average. These findings suggest a relationship between 1ATD and HFE mutations in the setting of cirrhosis. It was also described that the HFE H63D variant induces caspase activation and decreases cell proliferation associated with prolonged ER stress [47]. All these data are in agreement with our results and thus confirming our experimental pipeline.

HERPUD1, also named Herp, is a member of the ERAD pathway and it is involved through its direct interaction with the ubiquitin ligase HRD1 in the ubiquitination and retranslocation of ERAD substrates. Given our genomic screen results and the role of Herp into the ER, we subsequently focused our work on characterizing HERPUD1 variants effects in our 1ATD cellular model.

**Fig 4:**
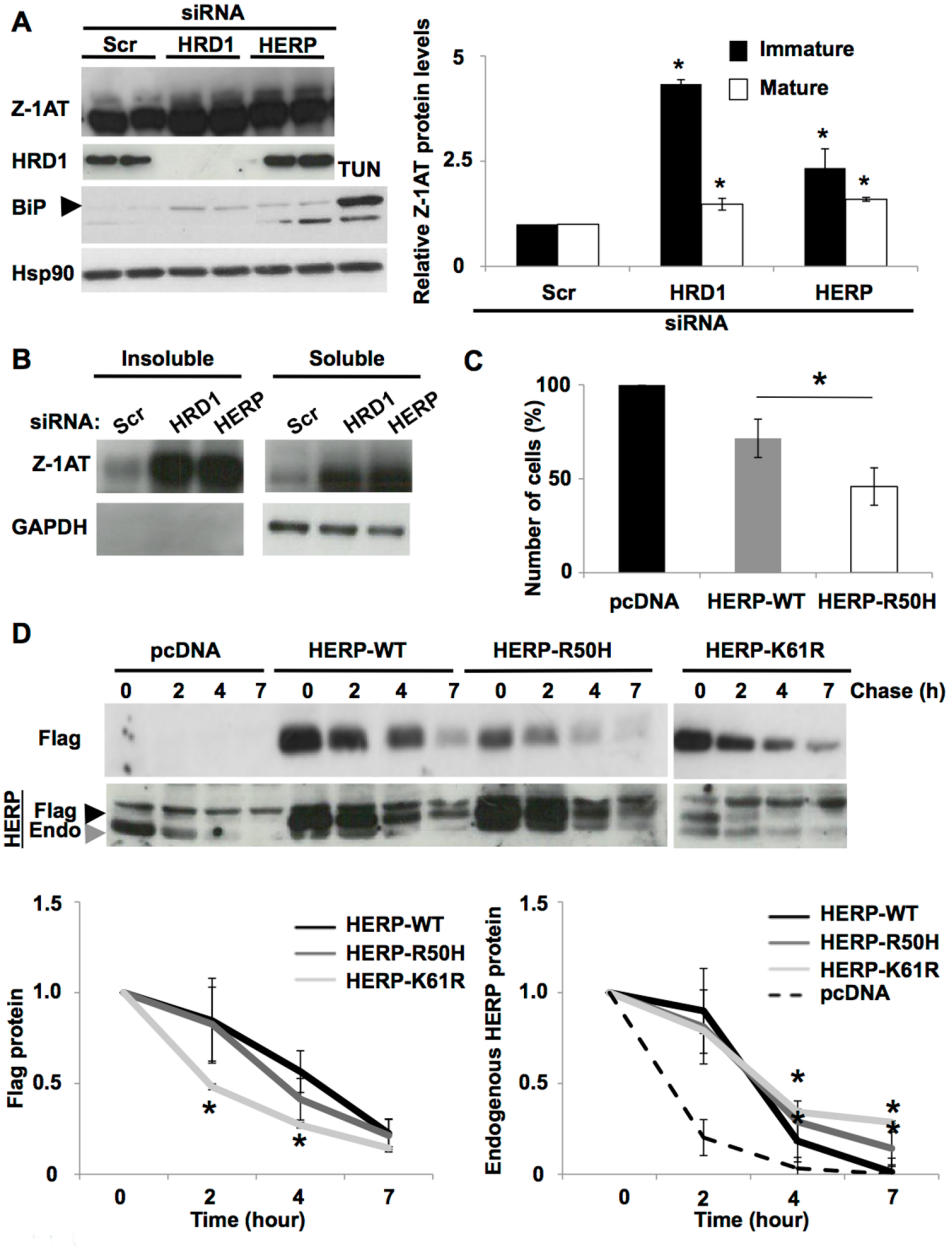
HERP-R50H stabilizes Herp endogenous and increases cell toxicity. (**A**) Immunoblot of 1AT, HRD1, BiP and Hsp90 protein expression in cell lysates following siRNA-mediated silencing of HERP and HRD1 in Z-IB3 cells. Tunicamycin (Tun) treatment at 2 μg/ml for 24 hours is used as positive control. Quantitative analyses of the immature (black bar graph) and mature (white bar graph) forms of Z-1AT after HERP and HRD1 silencing relative to scrambled control (Scr). Data denote the -fold change in the protein expression of the indicated Z-1AT forms relative to Scr (mean ± S.D., *n* = 3 independent experiments). In all panels, the * indicate *p* < 0.05 as determined by two-tailed t-test using Scr as reference. (**B**) Effect of HRD1 and HERP silencing on steady state levels of Z-1AT in Z-IB3 cells. Cell homogenates were separated into insoluble (left) and soluble (right) fractions and these fractions were then subjected to immunoblot analysis for 1AT (top) and GAPDH (bottom). (**C**) Percentage of live cells was estimated as described in Materials and methods upon pcDNA, HERP-WT or HERP-R50H overexpression at 48 h after transfection. Data are presented as a ratio relative to control pcDNA empty vector (mean ± SD, n = 3 independent experiments). The asterisk indicates p < 0.05 as determined by two-tailed t-test using pcDNA empty vector as the reference. (**D**) Immunoblot of Flag tag (upper) and HERPUD1 (Herp) protein expression (lower) in cell lysates following cycloheximide (CHX) treatment. Black arrowhead indicates Flag-Herp expression bands and grey arrowhead indicates endogenous (Endo)-Herp expression bands. Z-IB3 cells were transfected with plasmids encoding the empty vector (pcDNA), HERP-WT, HERP-R50H and HERP-K61R mutation. At 48 h after transfection, 50 μg/ml cycloheximide (CHX) were added and the chase was performed in the absence or presence of CHX for the indicated times. Quantitative analysis of the cycloheximide-chase experiments for the Flag tag (left) and Herp endogenous (right) upon pcDNA, HERP-WT, HERP-R50H or HERP-K61R overexpression is shown. Data shown denote the -fold change relative to *t* = 0 (mean ± S.D., *n* = 3 independent experiments). In all panels asterisks indicate *p* < 0.05 as determined by two-way ANOVA followed by Bonferroni post-test.

As it was already observed following HRD1 silencing (Fig 3B), Herp knockdown results in an increase of Z-1AT immature and mature forms produced and in a hyper activation of the UPR pathway (Fig 4A). In addition, we found that HRD1 and Herp silencing resulted in increased Z-1AT levels in both insoluble and soluble fractions (Fig 4B). These results are consistent and in agreement with the UPR hyperactivation observed following HRD1 and Herp silencing.

HERPUD1 is also a transmembrane ER protein with both amino- and carboxy-termini exposed to the cytoplasm. The amino-terminal HERPUD1 region contains a Ubiquitin Like Domain (UBL domain) from amino acid 10 to 81 [48]. Interestingly the HERPUD1 variant R50H, identified by WES, is located in this UBL domain. This domain has already been linked to its proteasome degradation and consequently its stability at the ER membrane. It has also been shown that either the deletion of the whole UBL domain or a K61R mutation in this domain, impair the degradation of the terminally misfolded truncated variant of 1AT, the Null Hong Kong (NHK) [23, 49]. This suggests a correlation between Herp stability, determined by the UBL domain, and its function in the ERAD process. Finally, Herp, which is strongly induced by the UPR [23, 50, 51] (Fig 3A and 3B), have an anti-apoptotic effect in the cellular response to ER stress [23, 49, 52, 53]. The ubiquitin-like domain of Herp is required for the survival of cells under ER stress [52, 53]. In brief, Herp is a short-lived ubiquitin-like protein improving the balance of folding capacity and protein loads in the ER and plays crucial roles for the ER stress fate.

Given the role of Herp in the degradation of other 1AT mutants and its effect on cell viability, we hypothesized that the mutant Herp-R50H identified by WES might destabilize its ERAD function and its protective effect on cell viability. To address this hypothesis, we first monitored cellular toxicity upon overexpression of the empty vector (pcDNA), HERP-WT and HERP-R50H (Fig 4C). We generated mammalian expression constructs containing the different variants of the human HERPUD1 cDNA. Equal amounts of the cDNA expression constructs were separately transfected into the human Z-IB3 cell line, and the toxicity was monitored. In agreement with previous reports [49, 52, 53], we observed a statistically significant decrease in live cells following the overexpression of HERP-R50H in comparison with the HERP-WT or the empty vector (pcDNA) (Fig 4C) in Z-IB3 cell line.

As mentioned above, the UBL domain of Herp was linked to its stability and consequently to its ERAD function [23, 49]. We then asked whether Herp-R50H might influence the protein stabilization. To address this hypothesis, Z-IB3 cells, expressing Flag tag HERP-WT, HERP-R50H and HERP-K61R were subjected to cycloheximide chase analysis. Unfortunately, overexpression of these different constructs was sufficient to induce a stabilization of those proteins at the ER due to the high expression level in our cell model (Fig 4D-top). To circumvent this difficulty we focused our study on the stabilization of endogenous Herp. Our different constructs: HERP-WT, HERP-R50H and HERP-K61R can be monitored by a change in migration on SDS-PAGE in response to the presence of the Flag tag. The slower migration of these different constructs allowed us to visualize and dissociate easily the expression of Herp endogenous (Fig 4D—Arrowheads grey and referred to as Herp Endo) from the expression of the others constructs (Fig 4D—Arrowheads black and referred to as Herp Flag). Remarkably, compared to the Herp-WT, Herp-R50H expression was shown to stabilize endogenous Herp (Fig 4D). The same result was observed for the well-characterized mutant Herp-K61R. Therefore, the single amino acid substitution R50H within the UBL domain seems to be sufficient to stabilize endogenous Herp at the ER membrane. Together our results suggest that Herp-R50H variant decreases ERAD efficiency by impairing its turnover at the ER membrane, consequently increasing the amount of misfolded proteins into the ER. This reduced efficiency compromises cell viability following the accumulation of the Z mutant and other misfolded proteins-mediated ER stress.

### ERAD impairment and HFE mutation H63D

In general and in a majority of patients, the Z-aggregates are tolerated by the hepatocytes. Conversely, we hypothesized that when ERAD machinery is impaired or slightly affected in cells that already accumulate Z-1AT aggregates, the global ER environment is affected and unable to deal with this minor perturbation. Then, this event increase the cell’s propensity to ER stress and finally to ER stress-mediated cell death.

**Fig 5:**
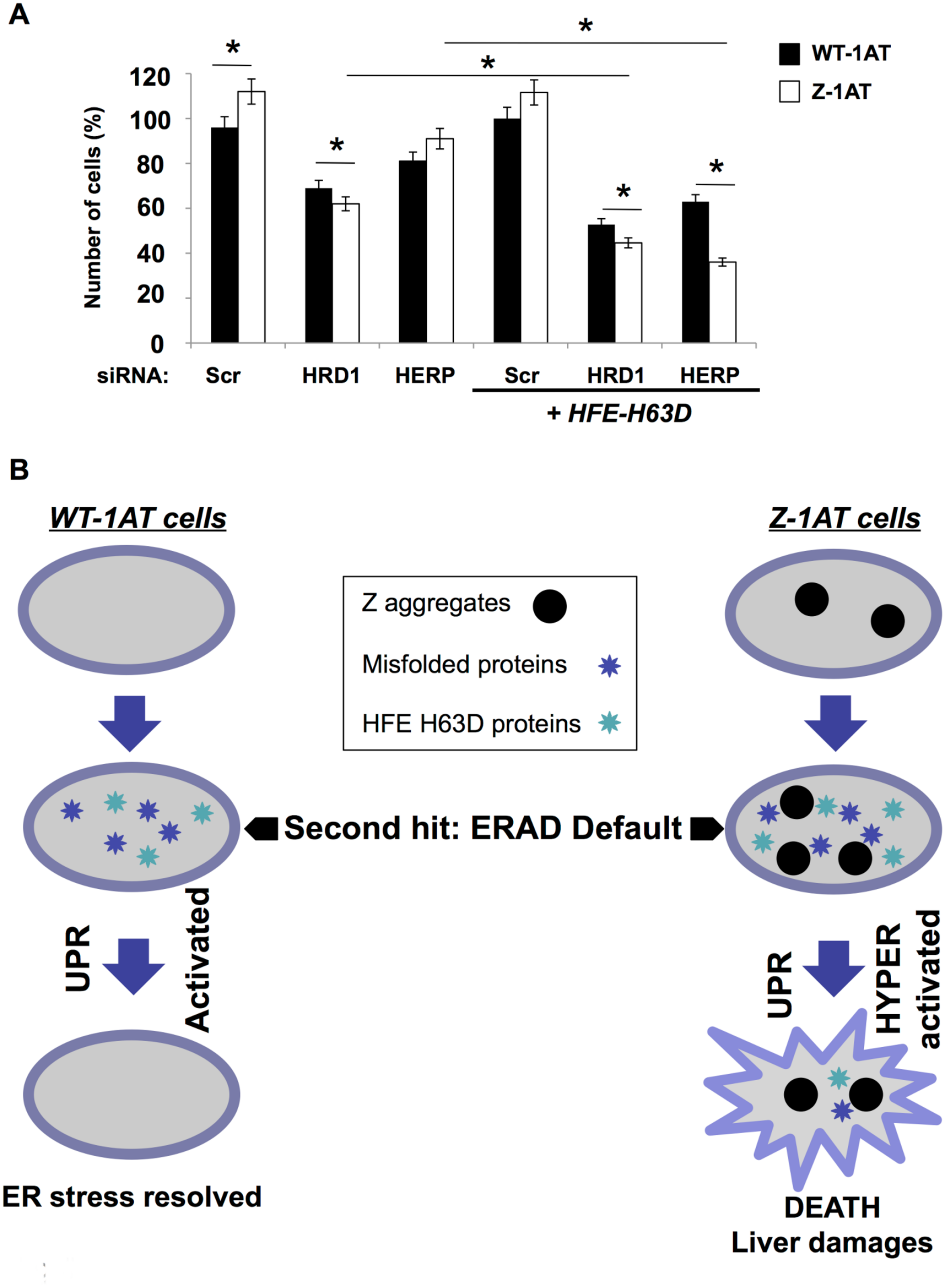
Combined effect of HFE-H63D variant and ERAD impairment in WT and Z-1AT expressing cells. **(A)** WT and Z-IB3 cells were treated with siRNA-mediated silencing of HERP or HRD1 for 72h and transfected with plasmid encoding HFE-H63D variant or pcDNA. At 48 h after transfection percentage of live cells for each conditions was determined. Data shown denote the -fold change relative to WT-IB3 cells (mean ± S.D., n = 3 independent experiments). In all panels asterisks indicate p < 0.05 as determined by two-tailed t test using WT-IB3 cells or siHERP Z-IB3 cells or siHRD1 Z-IB3 cells as reference. (**B)** Model for ERAD impairment and HFE-H63D mutation mediated liver toxicity associated with Z-1ATD. The model is adapted from Ordonez *et al* [42]. An insult resulting from the ERAD default increased the misfolded client proteins into the ER and this insult is effectively buffered by chaperone diffusion in WT-1AT cells. This prevents an UPR “hyperactivation” and the death of cells (‘‘ER stress resolved”). In contrast, in cells that accumulate Z-aggregates, the global ER environment is affected, which impairs chaperon access to misfolded proteins, thereby increasing the cell’s propensity to activate ER stress. An insult resulting from the ERAD default increased the misfolded client proteins, enhanced by the HFE-H63D mutation into the ER, and this insult is not effectively buffered by chaperone in Z-1AT cells. The cells are not anymore able to resolve the ER homeostasis (‘‘unresolved”) and therefore “hyperactivate” the UPR-mediated cell death.

To address this hypothesis, we first monitored the cellular toxicity following HRD1 and Herp silencing into WT-1AT or Z-1AT expressing cells. As previously shown in our study (Fig 2A and S2 Fig), we observed an increase of Z-1AT toxicity compared to WT-1AT following HRD1 silencing (Fig 5A). Interestingly, this difference of toxicity between the cells expressing the WT-1AT and the Z mutant was not observed following Herp silencing. This result is consistent with our WES data showing that patients who only carry the HERPUD1-R50H mutation do not develop major hepatic injury (Table 1).

Next, HFE H63D constructs were co-transfected into WT-1AT or Z-1AT expressing cells following the silencing of HRD1 or Herp. In our experimental conditions, a statistically significant difference in cellular toxicity was observed for the cells expressing Z-1AT compared to WT (Fig 5A) following the silencing of HRD1 or Herp and HFE H63D expression. This difference in cellular toxicity was also higher following the silencing of HRD1 or Herp + HFE H63D expression than upon the silencing of HRD1 or Herp alone. These findings suggest that ERAD impairment and expression of HFE H63D are associated with an increase in Z-1AT cellular toxicity, as suggested by our WES results (Table 1).

To further support our hypothesis, we performed WES on an additional homozygous Z-1AT family member (father, family 4) who had undergone lungs transplantation but did not present any liver dysfunction (Table 1). Remarkably, HERPUD1 SNP (rs2217332; c.G149A; p.R50H) was identified but not the H63D HFE mutation (rs1799945 c.C187G; p.H63D). Furthermore, in our small Z-1ATD families, the two identified SNPs cluster with liver damage severity. Both siblings with severe liver injuries (highly at risk of developing liver cirrhosis and cirrhosis/transplantation) carried these two SNPs (Herp R50H/rs2217332; c.G149A; p.R50H and HFE H63D/rs1799945 c.C187G; p.H63D) (Table 1), strongly suggesting their contribution to liver damages progression associated with 1ATD. All these data again support our hypothesis that ERAD defects and HFE variants are genetic modifiers for the severity and progression of 1ATD liver pathology component.

## Discussion

A single point mutation in Z-1AT is sufficient to induce the formation of liver aggregates that are retained as inclusions within the ER. This results in ER dysfunction and liver disease for 8% of the ZZ patients. Given recent evidences and based on the frequency of liver disease occurrence in Z-1AT patients, it is likely that modifier genes are linked to unfavorable liver disease progression. Based on our results, we predict that an ERAD dysfunction associated with a light or moderate accumulation of a misfolded protein such as HFE H63D overcome the liver’s capacity to deal with Z-1AT aggregates accumulation, which in turn trigger UPR mediated cell death and lead to a complete liver failure.

UPR pathway-mediated cell death has already been associated with 1ATD. Consistently with our study, it has already been shown that activation of the ATF6 pathway (one of the 3 branches of the UPR) limits Z-1AT-induced mitochondrial damage by promoting HRD1-dependent degradation of Z-1AT [54]. This result could explain in part the cell toxicity increase observed in our study. Moreover, it has already been shown that 1AT aggregate expression alone was insufficient to induce the UPR, but the resulting protein overload rendered cells hypersensitive to ER stress induced by either tunicamycin or glucose depletion [42]. The authors explained this difference by the fact that misfolded protein, in cells experiencing ER overload, could not diffuse freely, decreasing their accessibly to quality-control proteins required for folding and transport. This decreased mobility or availability of ER chaperones, resulting from changes in diffusive features and/or obstruction caused by protein overload, sensitizes the cell to subsequent activation of the UPR by a second hit that further increases the UPR burden.

Our result complete the model proposed by Ordonez *et al*. [42]. The expression of misfolded proteins as the mutant HFE-H63D is buffered in WT-1AT or Z-1AT expressing cells. This prevents dramatic pathways (apoptosis, necrosis…) induction and consequently severe hepatic injuries are not observed in ZZ patients carrying HFE-H63D variant (Fig 5B). A second insult represented here by an ERAD impairment (HRD1 silencing/Herp mutation) resulting in misfolded protein accumulation is effectively buffered by chaperone diffusion in WT-1AT expressing cells. This prevents ER stress and UPR activation (ER homeostasis resolved) (Fig 5B). Conversely, in cells that accumulate Z-aggregates, the global ER environment is already affected, thereby increasing the cell’s propensity to hyperactive the UPR pathway. Any additional impairment such as the ERAD dysfunction and the presence of other misfolded proteins (HFE H63D variant) is likely to overwhelm the system. In these conditions, cells are not able to resolve the ER homeostasis anymore and thus activate the UPR-mediated cell death (Fig 5B) and consequently liver damages.

Moreover, the mechanism proposed here could be relevant to other protein misfolding diseases, such as the serpinopathies or cystic fibrosis. In fact, some previously published work already shown that HFE mutations could modify disease severity in cystic fibrosis [55] as it was already observed for 1ATD [46].

In the light of a potential role for the UPR in the 1ATD-associated liver damages future studies will be necessary to further address and characterize the molecular basis of the UPR involvement in 1ATD. It will also be useful to screen for small molecule inhibitors of the different branches of the UPR. This idea is reinforced by a recent study that shows an activation of c-Jun N-terminal kinase (JNK) and c-Jun (a pathway induced by the IRE1 branch of the UPR) in model cell lines and in livers of patients homozygous for the Z allele [56]. The identification and validation of new pharmacological agents and drugable targets could open new therapeutic developments and offer the first medical treatment for patient with liver disease resulting from 1ATD.

Regarding of our results, we observed a rescue in traffic and secretion of the Z-variant following the silencing of HRD1 and HERP. These results could point to a promising therapeutic target to prevent emphysema associated to 1ATD. Unfortunately, as other before us observed [38, 49, 52, 54, 57, 58], silencing of the ERAD actors’ limits Z-1AT ERAD degradation, consequently increasing misfolded soluble and aggregate forms of Z-1AT usually leading to cell death.

Our results are also in agreement with previous studies that showed that Z-1AT has a different fate in iHeps derived from 1ATD patients with or without liver damages (Patient-specific iPSCs generated from 1ATD patients and differentiated into hepatocyte-like cells (iHeps)). These cells derived from 1ATD patients with liver damages, present especially a significantly slower Z-1AT intracellular degradation, than in iHeps derived from 1ATD patients without liver damages [59]. We suggest here that this difference could be explained by an ERAD dysfunction caused by HRD1 or Herp mutation. Our results highlight the role of the ERAD degradation machinery as a critical proteostasis mechanism for determining the severity of the liver disease, presumably by affecting the degree of proteotoxicity. Still, we cannot exclude that other cellular events are involved in this process. Two proteostasis pathways, ERAD and autophagy [26, 57, 60], have been shown to be implicated in the degradation of the ER-localized Z-1AT and could be involved in the proteotoxity associated with Z-1AT. It will be important in future studies to assess the implication of this latter pathway.

The first modifier gene identified, the endoplasmic reticulum mannosidase I (ERmanI), came from a candidate approach in a gene-sequencing study and suggested that differences in ERmanI expression were associated with an earlier age-of-onset of end-stage liver disease [11]. Our experimental pipeline did not identify any mutants of ERmanI but these results are not necessary incompatible since our sample size was limited and others groups have challenged the significance of this previous association [12].

Importantly, our conclusions will need to be confirmed in a larger group of affected ZZ patients before the definitive role of HERUD1 R50H and HFE H63D can be substantiated. However, these results support a larger scale screening for 1ATD and provide an exciting prospect for early prognosis and hold a considerable promise for innovative therapeutic interventions.

In either case, the results in this report take us one step closer to a possible identification of genetic factors linked with 1ATD-associated liver damages. Our findings are of a critical importance as currently, except liver transplant, no medical treatment is available. In the future, identification of additional genomic variants in HFE or any ERAD genes could consequently indicate at-risk patients for 1ATD-associated poor prognosis in liver disease progression. These patients would therefore be subjected to a more intense clinical monitoring and could even be placed on a liver transplant waiting list at an earlier time. In conclusion, our findings provide considerable promise for an early prognosis for 1ATD-associated liver disease and could even lead to possible therapeutic and pharmacologic developments.

## Supporting information

S1 Fig Yeast deletion strains with human ortholog. After our yeast screen only 5 deletion strains presented a selective toxicity for Z-1AT and had a clear human ortholog. The full list of candidates is composed by: COG6, MRPL1, MTOR and HRD1. Yeast gene RPD3 is closely related to the class I HDACs (HDAC1, 2, 3 and 8), we selected HDAC2 as it has already been involved in 1AT maturation and secretion [19].
(PDF)

S2 Fig Effects of candidate genes silencing on viability in models cell lines expressing WT or Z-1AT. The yeast screen provided us with a short list of candidate genes: COG6, MRPL1, TCO89 (MTOR), RPD3 (HDAC2/HD2) and HRD1. As MTOR is involved in two pathways in mammalian cells (MTOR complex 1 and 2) we tested an additional protein involved in MTOR complex 1: RPTOR. (**A**) Viability is measured using GF-AFC Substrate. This compound can enter live cells where it is cleaved by proteases to release AFC. WT and Z-IB3 cells were treated as described in Material and methods. Quantitative analysis of the effects of candidate genes silencing on viability in WT or Z-IB3 cells was plotted (mean ± S.D., n = 3). The * indicates *p* < 0.05 as determined by two-tailed t-test using WT-1AT as reference. (**B**) Luminescence, from a luciferase reaction, is monitored on WT and Z-IB3 cells, treated as described in Material and methods. Quantitative analysis of the effects of candidate genes silencing on viability in WT or Z-IB3 cells was plotted (mean ± S.D., n = 3). The * indicates *p* < 0.05 as determined by two-tailed t-test using WT-1AT as reference.
(PDF)

S3 Fig Effects of candidate genes silencing on traffic and secretion of 1AT in models cell lines expressing WT or Z-1AT. Immunoblot of 1AT and Hsp90 protein expression in cell lysates and culture media following siRNA-mediated silencing of the candidate genes in WT and Z-IB3 cells were quantified and plotted. The traffic of 1AT glycoprotein through the secretory pathway can be monitored by a shift on SDS-PAGE in response to the addition in the Golgi of ER-acquired N-linked oligosaccharides to the immature form (I) to generate the slower migrating, mature glycoform (Mat). The latter is then secreted by the cell to the serum (S). Quantitative analyses of all forms of WT-1AT (black bar graph) and Z-1AT (white bar graph) after candidate genes silencing relative to scrambled control (Scr) were plotted. Fold changes in the protein expression of WT or Z-1AT forms are indicated relatively to Scr (mean ± S.D., *n* = 3). The * indicates *p* < 0.05 as determined by two-tailed t-test using Scr as reference.
(PDF)
