# Supplementary material for: ERAD defects and the HFE-H63D variant are associated with increased risk of liver damages in Alpha 1-Antitrypsin Deficiency
Source: PLoS One. 2017 Jun 15;12(6):e0179369. doi: 10.1371/journal.pone.0179369 (PMC5472284; doi:10.1371/journal.pone.0179369)
Supplement: S1 Fig — After our yeast screen only 5 deletion strains presented a selective toxicity for Z-1AT and had a clear human ortholog. The full list of candidates is composed by: COG6, MRPL1, MTOR and HRD1. Yeast gene RPD3 is closely related to the class I HDACs (HDAC1, 2, 3 and 8), we selected HDAC2 as it has already been involved in 1AT maturation and secretion [19]. (PDF) [file pone.0179369.s001.pdf]

Gene	Description	Human ortholog
YNL330C/RPD3	*Histone deacetylase*	**HDAC2**
YNL041C/COG6	*Component of Oligomeric Golgi Complex 6*	**COG6**
YPL180W/TCO89	*Subunit of TORC1*	**MTOR**
YDR116C/MRPL1	*Mitochondrial ribosomal*	**MRPL1**
YOL013C/HRD1	*Ubiquitin-protein ligase*	**HRD1**

S1 Fig.
