# Supplementary figures and images for: ERAD defects and the HFE-H63D variant are associated with increased risk of liver damages in Alpha 1-Antitrypsin Deficiency
Source: PLoS One. 2017 Jun 15;12(6):e0179369. doi: 10.1371/journal.pone.0179369 (PMC5472284; doi:10.1371/journal.pone.0179369)

**A**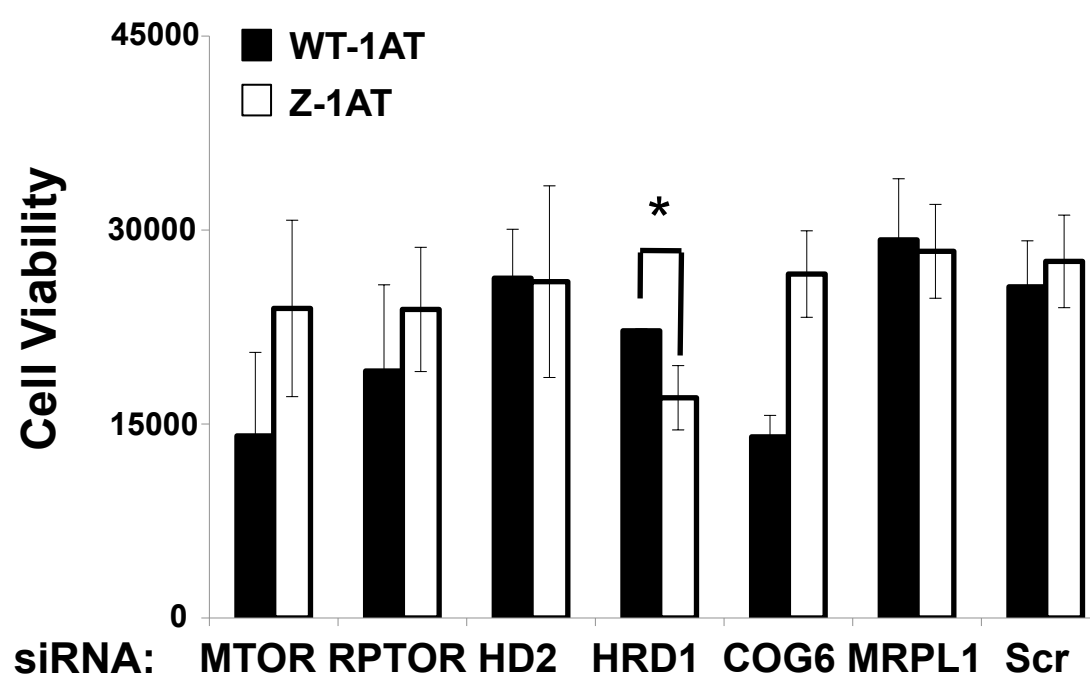**B**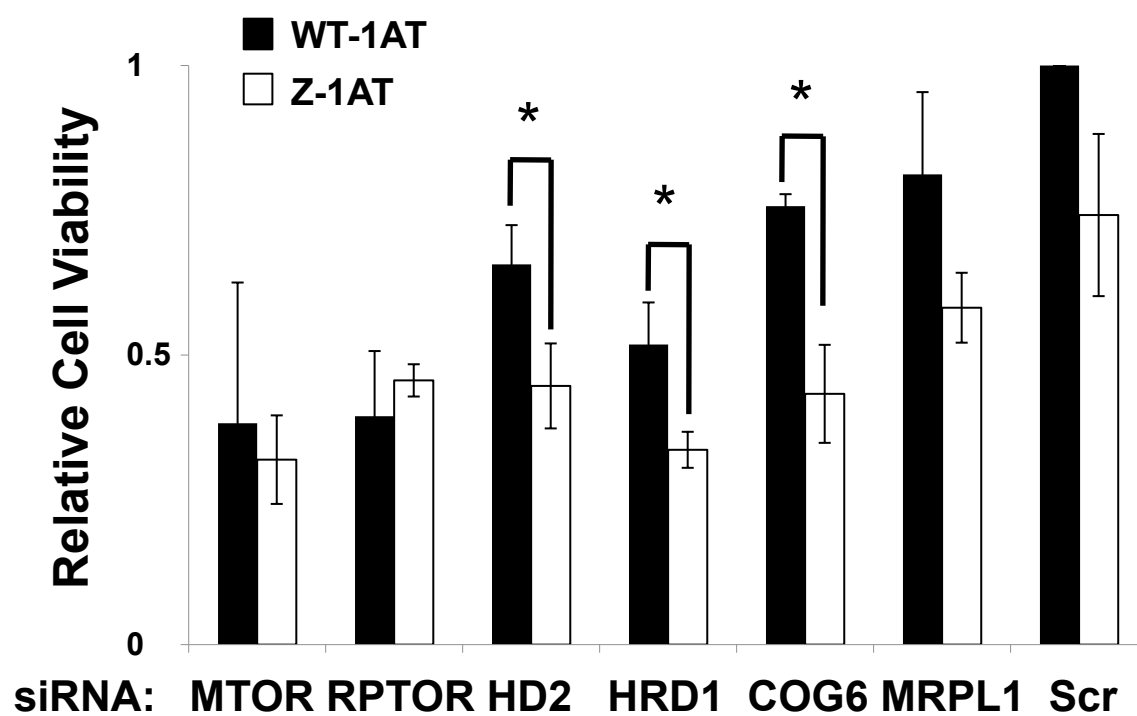**S2 Fig.**

Supplement: S2 Fig — The yeast screen provided us with a short list of candidate genes: COG6, MRPL1, TCO89 (MTOR), RPD3 (HDAC2/HD2) and HRD1. As MTOR is involved in two pathways in mammalian cells (MTOR complex 1 and 2) we tested an additional protein involved in MTOR complex 1: RPTOR. (A) Viability is measured using GF-AFC Substrate. This compound can enter live cells where it is cleaved by proteases to release AFC. WT and Z-IB3 cells were treated as described in Material and methods. Quantitative analysis of the effects of candidate genes silencing on viability in WT or Z-IB3 cells was plotted (mean ± S.D., n = 3). The * indicates p < 0.05 as determined by two-tailed t-test using WT-1AT as reference. (B) Luminescence, from a luciferase reaction, is monitored on WT and Z-IB3 cells, treated as described in Material and methods. Quantitative analysis of the effects of candidate genes silencing on viability in WT or Z-IB3 cells was plotted (mean ± S.D., n = 3). The * indicates p < 0.05 as determined by two-tailed t-test using WT-1AT as reference. (PDF) [file pone.0179369.s002.pdf]

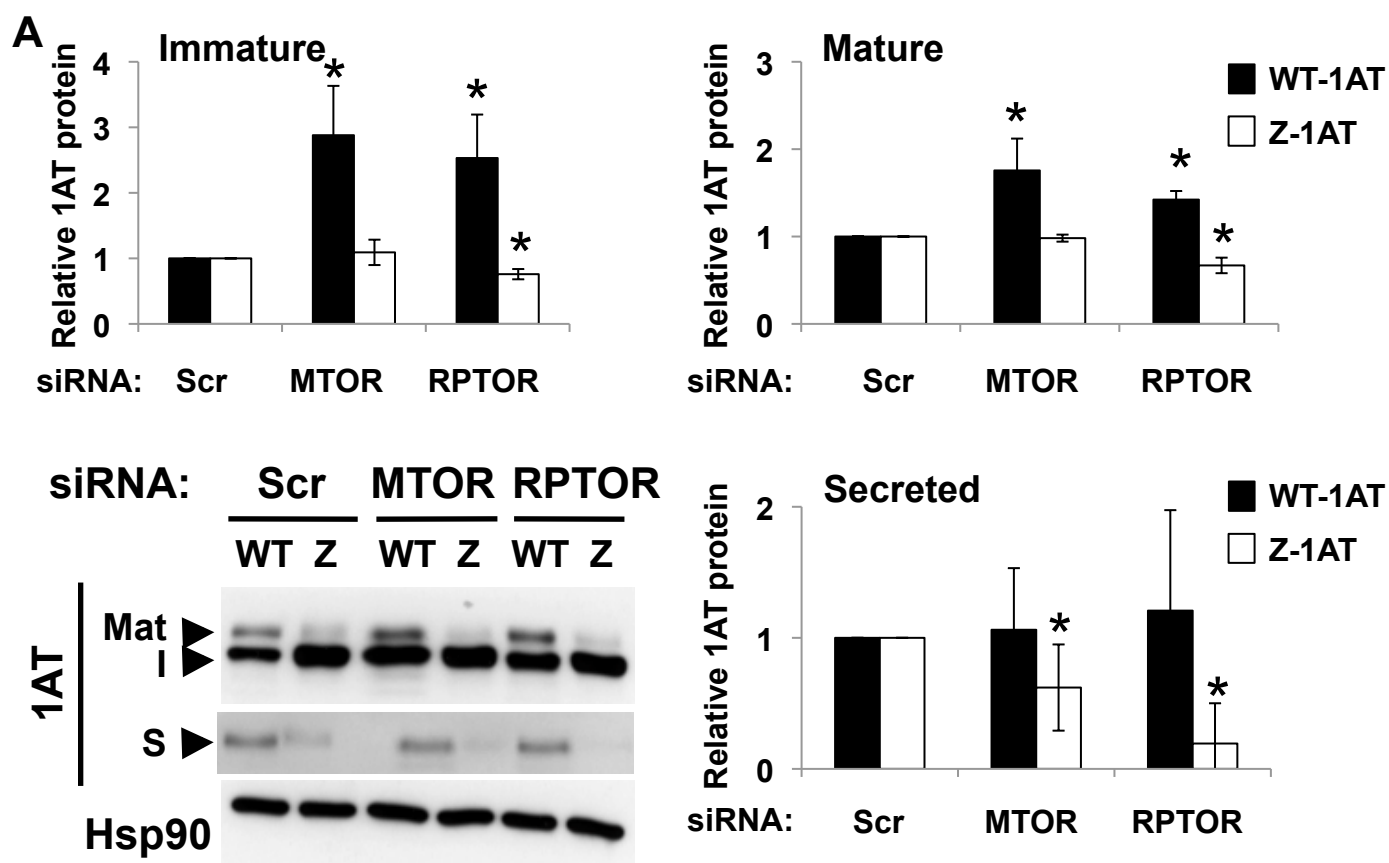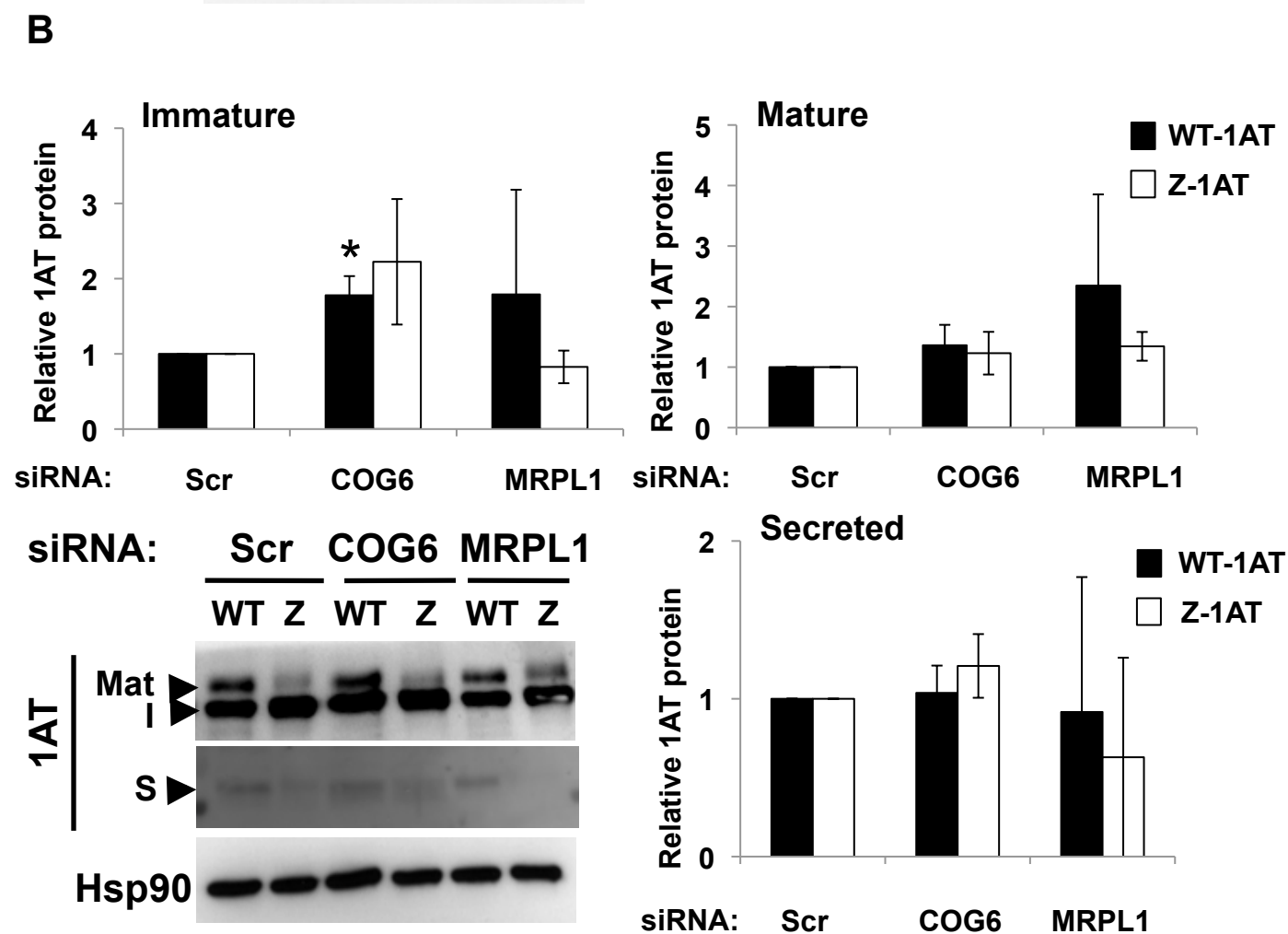

S3 Fig.

Supplement: S3 Fig — Immunoblot of 1AT and Hsp90 protein expression in cell lysates and culture media following siRNA-mediated silencing of the candidate genes in WT and Z-IB3 cells were quantified and plotted. The traffic of 1AT glycoprotein through the secretory pathway can be monitored by a shift on SDS-PAGE in response to the addition in the Golgi of ER-acquired N-linked oligosaccharides to the immature form (I) to generate the slower migrating, mature glycoform (Mat). The latter is then secreted by the cell to the serum (S). Quantitative analyses of all forms of WT-1AT (black bar graph) and Z-1AT (white bar graph) after candidate genes silencing relative to scrambled control (Scr) were plotted. Fold changes in the protein expression of WT or Z-1AT forms are indicated relatively to Scr (mean ± S.D., n = 3). The * indicates p < 0.05 as determined by two-tailed t-test using Scr as reference. (PDF) [file pone.0179369.s003.pdf]
